# Swallowing disorders in tracheostomised patients: a multidisciplinary/multiprofessional approach in decannulation protocols

**DOI:** 10.1186/2049-6958-9-36

**Published:** 2014-06-20

**Authors:** Giancarlo Garuti, Cristina Reverberi, Angelo Briganti, Monica Massobrio, Francesco Lombardi, Mirco Lusuardi

**Affiliations:** 1 Respiratory Rehabilitation, S. Sebastiano Hospital, AUSL Reggio Emilia, I-42015 Correggio, RE, Italy; 2 Neurological Rehabilitation, S. Sebastiano Hospital, AUSL Reggio Emilia, Correggio, RE, Italy

**Keywords:** Dysphagia, Swallowing, Tracheostomy cannula

## Abstract

Safe removal of tracheal cannula is a major goal in the rehabilitation of tracheostomised patients to achieve progressive independence from mechanical support and reduce the risk of respiratory complications. A tracheal cannula may also cause significant discomfort to the patient, making verbal communication difficult. Particularly when cuffed, tracheal cannula reduces the normal movement of the larynx which can further compromise the basic swallowing defect. A close connection between respiratory, phonating, swallowing and feeding abilities to be recovered, implies a strict integration among different professionals of the rehabilitation team. An appropriate management of tracheostomy cannula is closely connected with assessment and treatment of swallowing disorders in order to limit the development of severe pulmonary and nutritional complications, but at present there are no uniform protocols in the scientific literature. Furthermore, several studies report as an essential criterion for decannulation the presence of good patient consciousness, which is often altered in patients with tracheostomy, but a general agreement is lacking.

## Introduction

The positioning of a tracheostomy cannula following an acute episode of respiratory failure can help the management of patients who need long-term invasive mechanical ventilation, facilitating the aspiration of tracheal secretions, nursing and weaning from the mechanical support [1-3]. Once the subject is able to sustain spontaneous breathing, the primary steps of the rehabilitation programme include the safe removal of the tracheostomy cannula. An appropriate timing for removal must consider that:

1) spontaneous breathing has been re-achieved indefinitely

2) the risk of respiratory tract infection is reduced after restoration of the oropharyngeal barrier

3) an efficient cough is restored, re-establishing the function of the glottic plane

4) in case of a negative rehabilitation outcome, discharge to home or transfer to long-term care facilities are more difficult to implement in presence of a tracheostomy.

Unfortunately, in a significant percentage of patients with severe acquired brain injuries (23% in the paediatric population, 20% in adults) tracheal cannula cannot be removed or it must be repositioned after a removal attempt [4-9]. Awareness is necessary of the risks for the patient when the tracheostomy cannula has been removed [10], three aspects in particular must be evaluated before decannulation:

1) the anatomic patency of the airways,

2) the effectiveness of the defence mechanisms (particularly coughing),

3) the management of the patient’s oropharyngeal secretions and swallowing abilities.

Whereas the first two points are quite simple to assess with bronchoscopy, PCF (peak cough flow) and MEP (maximal expiratory pressure) measurements [11], the third item is more complex in terms of diagnosis and treatment.

## Review

Aim of this review is to discuss methods for the assessment of dysphagia in the context of decannulation protocols.

Methods for indentification of most relevant studies included the systematic search of the electronic databases MEDLINE and EMBASE using the following terms: dysphagia, tracheostomy, decannulation, swallowing evaluation test.

There is great variability in clinical practice, the lack of uniform behaviour and reference standards deriving from a limited number of studies in literature and limited dissemination of expertise among qualified personnel [12]. As a consequence, dysphagia is often underestimated and screening among the risk population is not an usual practice [11].

The presence of an endotracheal or tracheostomy tube, regardless of the basic disease, influences per se the laryngeal, phonation and swallowing functions [13-15], although recent studies do not always confirm such a close relationship [16,17]. Clinical experience has repeatedly shown that the removal of an endotracheal or a tracheostomy tube does not automatically restore the laryngeal function. Hoarseness and transglottic aspiration frequently follow tracheal intubation and tracheostomy [18,19]. An unprotected glottic plane is vulnerable to aspiration of secretions, food and gastric content, as it is to colonization from the oral flora. The frequency of swallowing disorders in tracheostomised patients varies from 50 to 83% depending on assessment methods, that are not standardized and have different diagnostic sensitivity [20]. Management must anyway be carried out by a multidisciplinary team.

## Swallowing in normal and pathological situations

### Physiology of swallowing

Swallowing is a complex neuromuscular process that enables the progression and transport of the bolus, either liquid or solid, from the oral cavity towards the lower digestive tracts. This action can be volitional, when eating, or reflexive following stimulation by saliva. It has been calculated that on average 590 swallowing actions are performed in a single day (145 during meals, 395 between meals when awake, 50 during sleep) [21].

Swallowing is classified into four successive and distinct phases, according to the anatomical region where the alimentary bolus is located:

1. Oral preparation

2. Oral phase

3. Pharyngeal phase

4. Oesophageal phase

The first two phases, during which the mylohyoid muscles contract rapidly, inducing swallowing to start, are under voluntary control. In the subsequent pharyngeal phase, the superior and middle constrictor pharyngeal muscles contract involuntary. The last, involuntary phase, concludes at the oesophagus with the contraction of the inferior pharyngeal constrictor muscle. In order to trigger this component by swallowing, afferent signals from the oral cavity converge in the spinal trigeminal system of the solitary fasciculus and lead to the swallowing centre in the nucleus of the solitary tract and in the reticular substance.

During the oral preparation phase the food is given a suitable consistency for swallowing. In the “*oral phase”* the tongue operates upward and backward movements, in a sequential compression and unrolling action towards the palate, hence pushing the bolus into the pharynx, through the coordinated, synergic and progressive action of the lingual, intrinsic and extrinsic muscles. The action of the tongue also has a predominant role in the elicitation of the following pharyngeal phase.

The “*pharyngeal phase”* takes place between the isthmus of the fauces and the part in front of the upper oesophageal sphincter. This is a critical moment in the swallowing process when the bolus moves across the aerodigestive crossroad [21]. Reflex stimulation of the pharynx leads to the release of the upper oesophageal sphincter, which allows the bolus to enter the oesophagus, starting the oesophageal stage. Immediately after, this sphincter contracts and closes again preventing oesophagopharyngeal reflux.

### Pathophysiology of swallowing disorders

During the “*oral preparation”* of the bolus, significant alterations may occur from different causes (see Table 1).

**Table 1:** Alterations that may be encountered in the oral preparation of swallowing

**Alteration**	**Consequence**
Reduced lip closure	Sialorrhea and food leaking out of the mouth
Limited jaw movements	Total or partial chewing disfunction
Lip, cheek and anterior 2/3^rd^ of the tongue sensitivity disorders	Pooled foods between cheeks and gums and difficulty in managing the bolus
Alterations in anteroposterior, lateral and vertical tongue movements	Difficulty in forming the bolus with possible falling and consequent aspiration
Reduced forward movement range of the soft palate	Possible leak of food into the pharynx and aspiration into the respiratory tract

In the “*oral phase”* there may be a reduced vertical or anteroposterior movement of the tongue or altered coordination with reduced propulsion and an increase in the oral transit time. Increased swallowing actions will be required to free the oral cavity from the bolus with a high risk of aspiration, being the respiratory tract still open.

As for the “*pharyngeal phase”*, a delay in swallow triggering leads to loss of control of the bolus, before the muscle activity preparing its entry into the pharynx. The viscosity, density and uniformity of the bolus affect the likelihood of its penetration into the respiratory tract. Liquid and inhomogeneous foods frequently facilitate aspiration, since they position at the glosso-epiglottic vallecula or in the piriform sinuses or straight in the larynx. Once the pharyngeal stage has been triggered, there may be various alterations to the neuromuscular events, as listed in Table 2.

**Table 2:** Alterations that may be encountered in the pharyngeal stage of swallowing

**Alteration**	**Consequence**
Soft palate failing to close	Bolus leaking out of the nasal tracts
Asymmetrical pharyngeal contraction	If the damage is bilateral the bolus will not progress on both sides and there will be pooled food
Reduced laryngeal range	Pooled food around the laryngeal opening and post-swallowing aspiration
Incomplete laryngeal closure	Aspiration and bolus pooling
Reduced laryngeal raising range	Bolus pooling and aspiration
Upper oesophageal sphincter dysfunction	Bolus blocking and possible returning into the respiratory tract

Swallowing defects differ according to the mechanism that has been compromised and may cause various symptoms (Table 3).

**Table 3:** Dysphagia classification, description of disorders and symptoms

**Type of dysphagia**	**Disorder**	**Symptom**
Neurogenic dysphagia in the vegetative state	Typical of patients who present a permanent vegetative state	
Neurogenic dysphagia from cognitive/behavioural deficit	Patients whose cognitive/behavioural deficits have a decisive effect on their ability to feed by mouth	
Neurogenic dysphagia for fluids	Patients are able to eat by mouth with a free diet	These patients present dysphagia for fluids and it is essential to introduce thickened liquids, with Aquagel, through parenteral or enteral therapy (NG-tube or PEG).
Mixed neurogenic dysphagia	Patients are not able to safely take more than one consistency	Patients who are fed with a semisolid diet and take thickened liquids, with Aquagel or through parenteral or enteral therapy (NG-tube or PEG) fall into this category.
Neurogenic dysphagia for solids	Patients are fed with a semisolid diet and fluids are administered by mouth	The subject cannot eat foods with a solid consistency due to inability to chew, difficulty in forming the bolus or inhalation. Dysphagia must be determined by a neurological and not a mechanical deficit.

## Decannulation protocol

For a successful decannulation process, swallowing evaluation must be combined with a pathophysiological study of respiratory function. The protocol should include several evaluations:

### Baseline oxygen saturation level (SaO_2_)

SaO_2_ must be over 92% breathing room air or with oxygen supplementation in patients with previous lung disorders, such as chronic obstructive pulmonary disease (COPD), in order to assure an appropriate tissue oxygenation.

### Need for mechanical aspiration

It can be assessed as number of tracheal aspirations over 24 hours; a cut-off is not established. Abundant bronchorrhea, and need for frequent aspiration are considered a relative contraindication to decannulation [22].

### Assessment of protective reflexes

This means in particular to evaluate the effectiveness of the cough reflex, by assessing the intensity of the cough either spontaneous or induced by tracheal aspiration. The absence of an effective cough is a contraindication to decannulation. A PCF over 160 L/min, eventually with adjuvant techniques such as manually or mechanically assisted cough, is in favour for decannulation [23].

### Chest X-ray

The presence of abnormalities at the chest x-ray, such as pneumonia or pleural effusion, may contraindicate decannulation.

### Fibrobronchoscopy

Essential to evaluate vocal cord mobility and tracheal patency. Vocal cord paralysis in adduction does not allow the patient to be decannulated.

### Capping the uncuffed cannula with SaO_2_ monitoring

This procedure aims to assess the patient’s ability to breathe through his/her own glottic plane; it also provides indirect information on tracheal patency. Few studies assessed the relationship between changes in SaO_2_ (measured noninvasively with a pulsoximeter) and aspiration. The results are contradictory but remarkable episodes of desaturation are associated with feeding in stroke patients [23-29]. One study highlighted SaO_2_ drops of 2% and 4% in 52% and 14% respectively of elderly people who did not present dysphagia [30]. Hence, arterial SaO_2_ variation may be attributed to several causes and there is currently insufficient evidence for direct correlation with dysphagia or aspiration.

In some cases the dimensions of the cannula are excessive for the tracheal lumen and when the cannula is closed the patient may experience difficult breathing. After checking the patency of the tracheal lumen with fiberbronchoscopy, the cannula can be replaced with a smaller one and the capping trial repeated.

The following statement was made at the III consensus conference on severe acquired brain lesions: ‘It is recommended to proceed with decannulation in subjects with a suitable level of consciousness, after clinical assessment of tolerance to the progressively longer capping of the cannula (up to at least 48 consecutive hours) and when the following criteria are met:

–SaO_2_ > 92% on breathing room air (FiO_2_ 0,21),

–effective cough with reduction in and/or ability to self-manage secretions,

–absence of infections,

–no significant abnormalities at the chest X-ray,

–at least partial swallowing effectiveness,

–absence of obstruction of the upper respiratory tract,

–satisfactory nutritional conditions’ [31].

## Assessment of dysphagia in tracheostomised patients

A dysphagia assessment protocol should include: 1. a detailed case history, 2. risk factor analysis, 3. tongue, mouth and face exercises, 4. evaluation of oral-nasal-pharyngeal secretion management, 5. swallowing tests, and 6. operating indications.

### Case history

“Personal data” and clinical diagnosis have to be recorded. *“Data on feeding modalities”* must be collected specifying if the patient is fed by mouth with a cuffed cannula or not. At admission, the patient should continue with the feeding method used during the previous hospitalisation step until a thorough assessment is made by the dysphagia expert team.

“Aspiration episodes” either actual or suspected should be registered. “Arterial desaturation episodes” are very important and precise circumstances when they might have occurred should be documented, such as during or after administration of meals, or during the decuffing of the cannula.

### Risk factors

The early identification of risk factors for dysphagia is particularly important, implying the assessment of: 1) vigilance, 2) pathological reflexes (e.g. bite, suction, trismus, snout reflex, bruxism, which could hinder or in some cases prevent the administration of the protocol); 3) presence of spontaneous swallowing; 4) presence of irritation reflexes; 5) possibility to maintain a suitable posture, 6) cranial nerve deficits, and 7) presence of voluntary and reflex cough [31-33].

**Figure 1:**
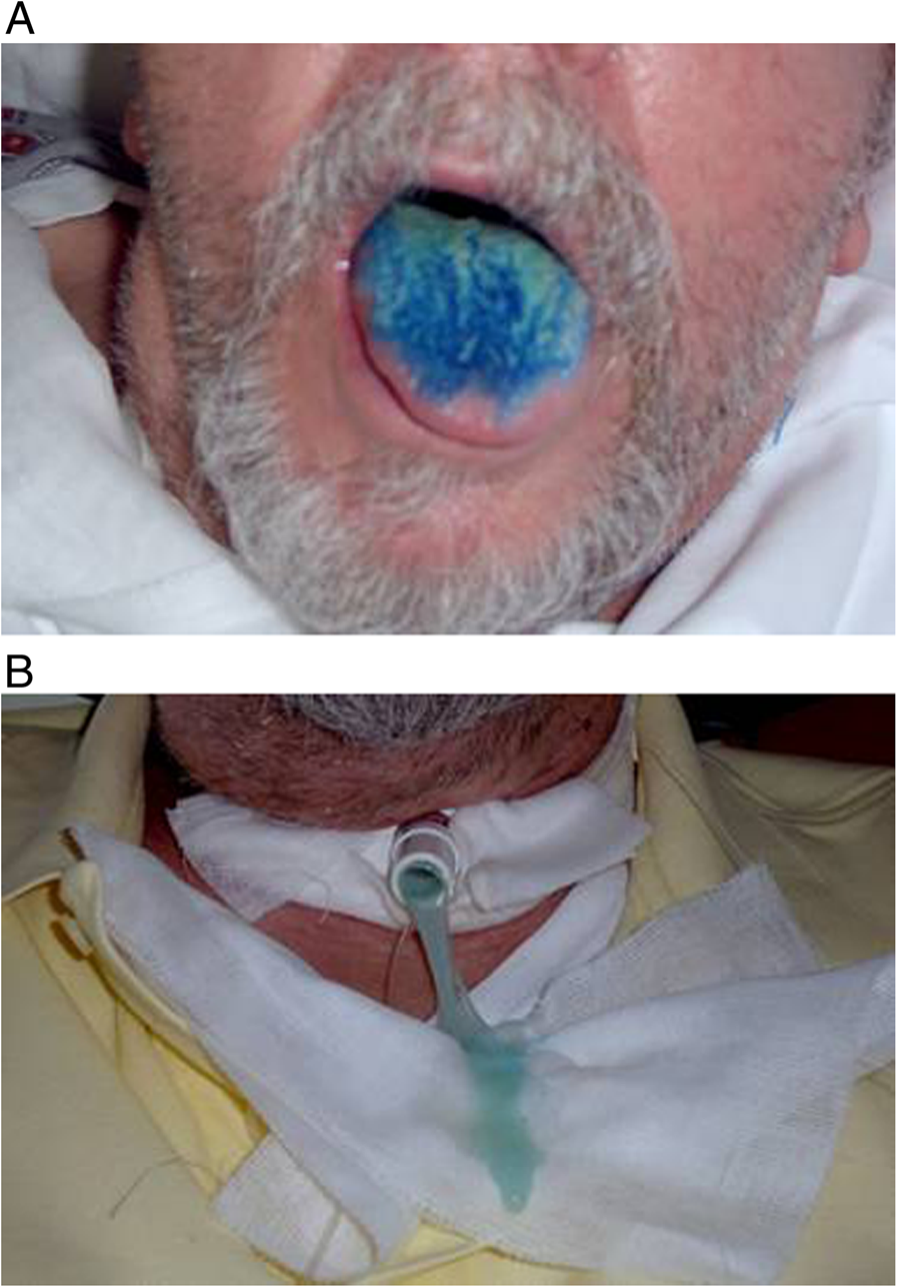
**Methylene blue swallowing assessment. A**. Oral administration of methylene blue. **B**. Appearance of blue colored secretions from tracheostomy in a dysphagic patient.

Before decannulation, an accurate evaluation of the effectiveness of the cough reflex is mandatory to prevent retention of secretions with an increased risk of infection and respiratory fatigue. Cough is an important alarm signal for inhalation; when elicitable, important indirect information can be obtained on the risk of aspiration. The presence of reflex cough must be evaluated during bronchoaspiration at different times of the day and in different postures. When cough cannot be elicited, silent inhalation may occur, with a potential risk of severe arterial desaturation and lower respiratory tract infection [34]. Silent inhalation can be easily demonstrated in a patient with a tracheostomy cannula, by colouring the secretions or food with methylene blue (Figure 1).

Cognitive, communicative or behavioural deficits must also be evaluated: inadequate focused and divided attention can be observed, with easy distractibility and exhaustibility. Significant behavioural or communication disorders (aphasia, dysarthria, apraxia) can occur, making it difficult to administer meals.

Patients with post-traumatic dysphagia or neurological disorders are often unaware of their own condition and are unable to control the consumption of food and/or adopt compensatory measures.

### Tongue, mouth and face exercises

The patient is asked to open the mouth, stick the lips out and smile; movement defects are noted, along with morphological alterations due to schisis or surgical operations. The patient is then asked to perform some tongue exercises to evaluate movement or strength limitations. To follow, the velopharyngeal sphincter is assessed, by asking the patient to puff out his/her cheeks; any air leakage through the nose can be checked for by placing a small mirror under the patient’s nose. Finally, the velar function at rest and during phonation is assessed, recording any raising deficits or inadequate seal. The exercises are carried out following verbal instructions and/or by imitation; if the patient has trouble with the voluntary performance of movements that on the contrary are carried out correctly automatically or as a reflex, the presence of buccofacial apraxia can be supposed.

After risk factor and objective evaluation two general considerations can be derived:

–it is reasonable to begin nutrition by mouth only in conscious and cooperative patients.

–food must be administered with great caution in patients who are unable to open their mouth, stick out their tongue or lack volitional control of reflexes protecting the respiratory tract such as coughing and throat clearing. In some cases, however, a careful assessment can allow the speech therapist to start administering small amounts of food for rehabilitation purposes (taste stimulations).

### Evaluation of oral-nasal-pharyngeal secretion management

A cuffed tracheostomy cannula must be deflated, to assess the spontaneous management of secretions; SaO_2_ variations or respiratory symptoms must be recorded.

Quite a common procedure at this stage is to colour the secretions with methylene blue. A few drops are put into the mouth and the patient is asked to swallow; after a few swallowing actions the coughing reflex is checked for with methylene blue coming out of the tracheostomy cannula (immediate inhalation). Bronchoaspiration is performed to check for any methylene blue in the trachea (aspiration). The test is normally carried out at least twice in the same day, asking the healthcare personnel to report any presence of methylene blue in the secretions spontaneously removed by the patient or during mechanical aspiration (late inhalation). SaO_2_ monitoring is recommended throughout the test. In presence of signs of inhalation, the clinical relevance must be defined by instrumental data (eg, severity and duration of desaturation); if the inhalation is mild, there is no arterial desaturation and there are no other signs of respiratory complications (cough, chocking etc), the assessment can be continued; at the same time, it is possible to start keeping the cannula deflated for increasing periods (first during the day, then at night) checking for episodes of arterial desaturation and occasional or recurring lower respiratory tract infections. If the inhalation of saliva takes place in the absence of such two complications, the swallowing assessment protocol can be performed for possible decannulation. Evidence of saliva inhalation is *per se* significant, implying a specific risk for the patient, but not always of clinical relevance, since dysphagic patients can be observed with inhalation of saliva demonstrated with methylene blue but without episodes of arterial desaturation or pulmonary complications [35]. They are usually patients who maintain significant cough effectiveness and can protect themselves from inhalation. In these cases, after a suitable period of clinical observation with the decuffed cannula, it is possible to proceed with the decannulation protocol. On the contrary, if the inhalation of secretions occurs with frequent episodes of desaturation or recurring infections of the respiratory tract, it is necessary to keep the cannula cuffed and in some cases to abandon the weaning protocol.

### Swallow tests

The swallowing assessment with methylene blue is an important method to detect any form of dysphagia before decannulation [31,35].

The presence of the cannula makes the assessment easier to be made (particularly to identify silent inhalation) and the dysphagia to be treated (with or without food) with relevant safety for the patient.

#### Ability to swallow fluids

10 mL of water with methylene blue are administered, then 50 ml. This test assesses, in particular:

1) The oral preparation phase which, in the event of swallowing fluids, comprises the prehension ability of the lips and their strength, the presence of lip continence, tongue grooving and the oral control of the fluid.

2) The propulsive oral phase which includes the anteroposterior movement of the tongue and the maintenance of suitable mouth muscle tone to prevent the fluid falling into the glosso-epiglottic vallecula.

3) Assessment of the pharyngeal reflex triggering.

Breathing-swallowing coordination must be evaluated; if there are signs of coordination defects, the activation of defence mechanisms must be verified.

It is also necessary to observe whether there is a normal laryngeal movement considering that the presence of the tracheostomy cannula represents *per se* a mechanical obstacle to larynx raising. In this observational test, a semi-quantitative scale should be used that measures the impairment of these stages.

4) The presence of the premature falling of the fluid and indirect signs of penetration (gurgling voice) or inhalation in the nasal tracts (nasal regurgitation) should be observed.5) The presence of reflex cough must be verified with methylene blue coming out of the tracheostomy cannula (immediate inhalation). It is important to assess whether reflex cough presents before (pre-swallow cough), during (intra-swallow cough) or after swallowing (post-swallow cough). The “quality” of the reflex cough must also be ascertained, i.e. whether it is effective, weak or gurgling. The absence of reflex cough can be interpreted both as a positive sign (if at the subsequent bronchoaspiration there are no traces of methylene blue) or as a negative prognostic sign of silent inhalation (if at the subsequent bronchoaspiration there are traces of methylene blue). Figure 1A and B.

The liquid swallow test is normally carried out at least twice in the same day, asking the healthcare personnel to report any presence of methylene blue spontaneously eliminated by the patient or during aspiration (subsequent inhalation). During the test it is useful to monitor SaO_2_.

#### Ability to swallow semisolid foods

This test must be carried out on a different day from the previous test so as not to confuse early symptoms with possible late consequences due to the inhalation of liquids. A semisolid food with methylene blue is administered by the speech therapist using a spoon.

The same data as for the liquid assessment are collected, with the addition of the presence of residuals inside the oral cavity.

The same procedures should also be followed: the test is performed at two different times of the day and the personnel are asked to report any presence of methylene blue spontaneously eliminated by the patient or during aspiration (subsequent inhalation).

#### Ability to swallow solids

This is performed only if the patient does not present inhalation for fluids and semisolid foods.

If the patient only presents dysphagia for fluids, a semisolid diet is recommended for a few days and then the swallowing ability is assessed for solids. The assessment of solids is only performed after checking that the patient is able to eat a full meal without any particular difficulty.

In general the same assessment protocol is used, administering a food with a solid consistency (for example, pasta, bread, biscuits, etc.). During and at the end of the administration, bronchoaspiration is performed to check for inhalation.

After exclusion of dysphagia, it is possible to proceed with the decannulation protocol; if the patient is dysphagic, there are two possibilities:

–If dysphagia can be resolved in reasonable timescales (4-6 months), decannulation can be postponed if maintaining the cannula is believed to be useful for rehabilitation purposes

**Figure 2:**
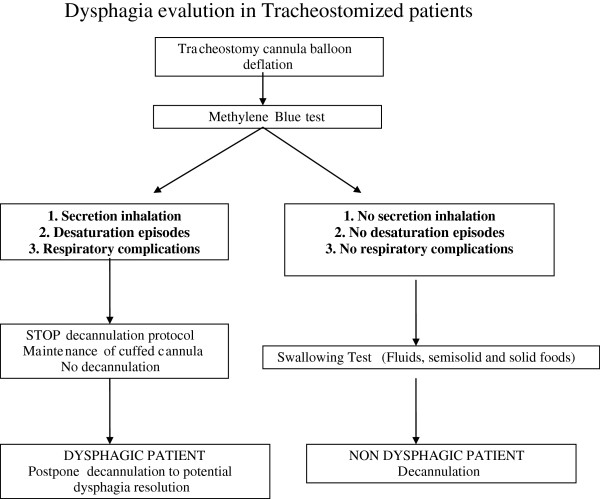
Flow chart for decannulation of tracheostomised patients in reference to swallowing disorders.

–If dysphagia is severe and not likely to be resolved in one-two months, the patient can be decannulated anyway, postponing nutrition by mouth until the patient has improved neurologically.Figure 2 reports a flow chart for patient decannulation in reference to swallowing disorders.

### Operating indications

After assessing the oral-nasal-pharyngeal management of secretions and the swallow tests, the indications on the patient are recorded in a written report accessible to physician, nurse and caregivers.

The report indicates whether: 1. speech therapy is proposed or it is necessary to make a systematic observation; 2. the patient can be fed by mouth and the consistencies allowed (free diet, semisolid or semi-liquid diet); 3. fluids can be administered by mouth; 4. the patient must be fed under supervision and who must supervise (speech therapist, professional nurse or technical operator, relative etc.); 5. to start administration of taste stimulations by the speech therapist, in case the patient is not fed by mouth; 6. finally, the consultancy of the speech physician is required and a videofluoroscopic swallow evaluation is recommended.

## Instrumental assessment

The clinical instrumental assessment must establish the integrity of the structures involved in swallowing, as well as the physiological operation of the oral effectors, pharynx, larynx, cervical oesophagus during the passage of the bolus.

The clinical indication for an instrumental assessment is necessary if the screening procedures (particularly Bedside Swallowing Evaluation, B.S.E.) are not exhaustive [36] expecially if there are any compromised neuropsychological abilities and the patient is at high risk of dysphagia. Instrumental evaluation can be postponed in case of unstable clinical conditions (such as compromised respiratory gas exchange), lack of cooperation or if the treatment plan cannot be changed [37,38].

The instrumental assessment allows a diagnostic definition of the symptoms [28] and a clinical severity stratification by documenting the progression of secretions or bolus in the lower respiratory tract or penetration (progression to the vocal cords) and inhalation (progression below the vocal cords) [39-41].

At the moment, there is no instrumental gold standard to predict complications in patients with dysphagia [42]. Endoscopic and radiological evaluations with a dynamic study and video recording can be considered equivalent in the assessment of swallowing [43,44].

Both have similar sensitivity, specificity and predictive value in identifying antegrade aspiration [45].

The videofluoroscopic swallowing study (VFSS) or other digital investigations, with the method of the “modified barium swallow” (MBS) [36-38] allow the whole swallowing action to be studied, from the oral cavity to the stomach, without any information on the sensitivity of the individual districts.

Fiberoptic endoscopic evaluation of swallowing (FEES) [46-48] uses a nasopharyngoscope introduced into the pharyngeal cavity through the nasal fossa. It allows the pharyngeal stage of swallowing alone to be studied, obtaining indirect information on the oral and oesophageal phases. The FEES enables an elective study of the laryngeal sphincter, the sensitivity and the display and management of secretion retention to be performed. It is less invasive than bronchoscopy and can be carried out in bed even on patients with unstable conditions [49].

It should preferably be performed by an otolaryngologist [50] or health personnel appropriately trained on swallowing pathophysiology and rehabilitation.

Other dynamic methods are less common. They include:

–FEESST (Fiberoptic Endoscopic Evaluation of Swallowing with Sensory Testing), combining the endoscopic evaluation with a study of sensitivity by delivering air pulse stimuli [51]

–Manofluorography, combining radiological evaluation with the detection of pressure changes of the pharynx when the bolus passes [52,53]

–Scintigraphy, assessing the progression of the bolus marked with a radioactive tracer (quantitative assessment) [54]

–Cervical auscultation, carried out using a phonendoscope to listen for gurgling. The technique is still to be re-assessed in terms of clinical trials [55,56].

### Diagnostic labels of dysphagia

Since dysphagia may derive from multiple causes its definition varies according to the clinical status of the patient. Diagnostic labels can be classified as follows [57]:

1) *Neurogenic dysphagia in the vegetative state*: typical of patients in a permanent vegetative or minimally responsive state.

2) *Neurogenic dysphagia from cognitive/behavioural deficit*: patients are unable to eat by mouth for cognitive/behavioural problems.

3) *Neurogenic dysphagia for fluids*: patients are able to eat by mouth with a free diet, but dysphagia for fluids prevents them to drink. In these patients it is essential to introduce thickened liquids, with aquagel by oral administration or through parenteral or enteral therapy by NasoGastric (NG) or Percutaneous Endoscopic Gastrostomy (PEG) tubes.

4) *Mixed neurogenic dysphagia*: patients are not able to safely take more than one consistency; for example, patients who are fed with a semisolid diet and take thickened liquids, with Aquagel.

5) *Neurogenic dysphagia for solids*: patients are fed with a semisolid diet and fluids are administered by mouth; the subject cannot eat foods with a solid consistency due to chewing inability, or difficulty in forming the bolus or inhalation.

Dysphagia must be caused by a neurological and not a mechanical deficit: i.e. patients who cannot eat solid food only because they are not wearing a dental prosthesis do not fall into this category. In other words, it must be clear that the onset of the neurological deficit has led to the loss of a function that was normal before.

In some cases, patients may be classified according to more than one type of dysphagia (for example mixed and cognitive/behavioural dysphagia); in these cases the type of dysphagia is labeled according to the prevalent disorder.

As well as the definition of the dysphagia, the severity rating should also be reported, i.e. the DOSS (Dysphagia Outcome and Severity Scale) [58].

## Conclusions

The removal of the tracheostomy cannula is an important rehabilitation goal, but cannot always be performed [59,60]. As a matter of fact, decannulation is a complex and multidisciplinary process, which considers various aspects from cognitive to critical issues such as protecting the respiratory tracts. Swallowing represents a fundamental aspect in this process. There are currently few documents that indicate shared protocols for the assessment of swallowing in the decannulation process. The final document of the recent Consensus Conference on severe acquired brain injuries promoted by the SIMFER (Italian Society of Physical Medicine and Rehabilitation) [31] represents a summary of the various experiences acquired in Italy which could apply not only to severe brain injuries but also other disorders. Studies on this aspect are also lacking. A trial was recently presented in which for 54 patients who had concluded the protocol as indicated by the SIMFER recommendations, decannulation was possible in 42 out of 54 cases (77.9%). Inability to manage saliva and dysphagia represent the main reasons that slow down or do not permit the implementation of the decannulation project, although they do not represent an absolute contraindication to decannulation. Another retrospective study shows how a multidisciplinary approach with a swallowing assessment can lead to a high decannulation percentage (99.5%), even more quickly (48 days after insertion) compared to an approach without a multidisciplinary protocol (88% with 94 days interval from insertion to decannulation) [61]. However, the severity of the clinical and neurological state seems to have a significant influence on decannulation failure [62].

Decannulation is also possible in selected cases of patients in a vegetative or in a minimally conscious state after verifying a reasonable effectiveness of cough and spontaneous swallowing. In any case, rehabilitation of patients with a tracheostomy cannula requires a close integration among the various professional figures with a particular regard to the assessment of the dysphagia.

## Competing interest

The authors declare that they have no competing interests.
